# A Comparative Study of Cardioprotective Effect of Three Anesthetic Agents by Measuring Serum Level of Troponin-T after Coronary Artery Bypass Grafting

**Published:** 2012-09-15

**Authors:** Vali Imantalab, Abbas Seddighi Nejad, Ali Mir Mansouri, Alimohammad Sadeghi Meibodi, Mohammad Haghighi, Heidar Dadkhah, Mohammadreza Mobayen

**Affiliations:** 1 Department of Cardiac anesthesia, Rasht University of Medical Sciences, Rasht, IR Iran; 2 Department of Burn Surgery, Motahari Burn Hospital, Rasht University of Medical Sciences, Tehran, IR Iran

**Keywords:** Cardiac Surgery, Troponin T, Ischemia Preconditioning, Isoflurane, Propofol, Midazolam

## Abstract

**Background:**

Cardiac surgery is associated with some degree of myocardial injury. Preconditioning first described in 1986 was pharmacologic and non- pharmacologic. Among the long list of anesthetic drugs, isoflurane as an inhaling agent along with midazolam and propofol as injectable substances have been documented to confer some preconditioning effects on myocardium.

**Objectives:**

In this study cardiac Troponin T (cTnT) ,as a reliable marker, was used for evaluating myocardial injury.

**Methods:**

This prospective double blind study was comprised of 60 patients scheduled for CABG and were randomly assigned into three groups who received infusion of propofol or midazolam or isoflorane. Surgical procedures and anesthetics were similar for 3 groups. cTnT measured preoperatively and at 12, 24 and 36hr after arrival in ICU.

**Results:**

There were no statistically significant differences in mean cTnT levels between three groups in the preoperative period and 12-24 hours after arrival in ICU. However, mean cTnT in 3 groups at 36 hours after arrival in ICU were different (*P*< 0.013) and cTnT level was significantly higher in midazolam group (*P*<0.001) and lowest in isoflurane group (*P*=0.002).

**Conclusion:**

There were significant differences on cTnT levels between anesthetic groups of isofluran, midazolam and propofol at 36 hr after surgery. Preconditioning effect of isoflurane was higher than the other two groups.

## Introduction

Despite advances in surgical treatment of coronary artery disease (CAD), due to the newer techniques and anesthetic protocols held in coronary artery bypass grafting (CABG), perioperative myocardial damage still remains as an unavoidable threat (1-3). To select the most reliable marker of cell damage, cardiac Troponin (cTn) is found to be the most sensitive and specific marker in diagnosis of myocardial necrosis (4,5) among which cTnT has already received a huge interest (6).

By using a combination of agents, anesthesia aims to maximize its effects while avoiding adverse events (5,6). However, studies have discussed the role of anesthesia in imposing additional ischemic stress on the myocardium already burdened with ischemia (7). On the other hand, protective effect of anesthetic agents on myocardium has received much attention either by cardiac surgeons or anesthetists (8-11). As proposed earlier by Murry et al. in 1986 (12), contemporary myocardial ischemia could potentially protect cardiac muscle from further damages; a process which was later called preconditioning (12,13).

Among the long list of anesthetic drugs, isoflurane as an inhaled agent along with midazolam and propofol as injectable substances have been documented to confer some preconditioning on myocardium (8-10,14,15) Although studies have evaluated the cardioprotective effects of anesthetic combinations on myocardial ischemia, there is no compelling evidence to compare the level of preconditioning evoked by either of these agents in relation to the extent of cell damage measured by serum cTnT concentrations. (16)Therefore the present study was carried out to secure these objectives in an academic hospital setting.

## Materials and Methods

This prospective study was a randomized clinical trial and carried out by the Department of anesthesiology of Guilan University of Medical Sciences Rasht, IR Iran. A total of 60 consecutive patients eligible for elective CABG were enrolled. Helsinki declaration statements were followed during the study. The research protocol was approved by institutional review board of the hospital. The patients also filled an informed consent before entering our study. The patients excluded from the study were those with ejection fraction (EF) lower than 40%, or undergoing redo or emergent CABG, concomitant valve procedure or aneurismal surgery, experiencing unstable angina, having left bundle branch block (LBBB), consuming oral hypoglycemic agents or theophyline, having implanted pace maker, or preoperative administration of inotropic agents, and single or two vessels diseases.

All patients had three vessels disease and CABG was performed by two surgeons using the same technique. Patients were randomly allocated into three groups of anesthesia including those receiving propofol, or isoflurane or midazolam. All patients were visited by anesthesiologist and then gave blood sample for a baseline serum level of cTnT the night before surgery and were administered 0.1 mg/kg intramuscular morphine 1 hour before anesthesia. The procedures carried out in the operating room (OR) were cardiac monitoring by 5 leads electrocardiography (EKG), pulse oxymetry, left radial artery catheterization, access to central venus through cannulation of right jugular vein, capnography, central temperature monitoring through nasopharynx, checking urine output, and examination of bispectral index. Anesthesia was induced by slow injection of sufentanil 1µg/kg intravenously followed by infusion of 0.2 mg/kg etomidate, and 0.15mg/kg cisatracurium was also infused for muscle relaxation. Anesthesia was maintained by three different agents; either by 0.2 µg/kg/h midazolam, by 6-8 mg/kg/h propofol, or by inhaling isoflurane.

Lung ventilation was controlled with 100% oxygen and adjusted respiratory rate. Activated clotting time( ACT) was also measured following administration of 300 μ/kg heparin and cardiopulmonary bypass was initiated if ACT> 480 seconds. Hemodynamic status was stabilized by using epinephrine or norepinephrine and intravenous fluids determined by standby anesthetist.

Cardioplegic solution was administered antrogradely along with standardized regional and systemic hypothermia at 30-32C°. Revascularization was performed with harvested saphenous veins and internal mammary arterial grafts. Following normothermia and maintaining heart rate in normal range, patient went off- pump and ventilation re-established.

After the surgery, patient was transferred to the intensive care unit (ICU) with resuming ventilation and close observation. The criteria met before patients were extubated and ensuring that cardiovascular and respiratory status are stabilized included hemodynamic stability, blood loss less than 120 ml per hour, core temperature > 36 °C, as well fully answerable or oriented status. cTnT was measured by obtaining blood samples at 12 h, 24 h, and 36 h after arrival in ICU, employing enzyme linked immunosurbant assay (ELISA) (Kit Troponoin T Elecsys 2010 made in France) while the values were deemed detectable at a lower limit of 0.01 ng/ml. All patients in ICU received the same care, medication and comparable interventions.

Demographics and operating data were recorded in association with laboratory results on serum levels of cTnT at different time intervals from anesthesia. Data were analyzed, using Statistical Package for Social Sciences (SPSS, version 18, Chicago, Inc), to compare the relationships between the levels of cTnT and anesthetic agents. Chi square and cross tabulation were employed for qualitative variables while one- way analysis of variance and general linear model repeat measurements were applied to analyze quantitative variables. A P value of< 0.05 was considered statistically significant.

## Results

Demographic characteristics of patients including sex, age, BMI (Body mass index) (Table1), and coexisting diseases revealed no significant differences between the three groups (Table 2). Also no differences were found in regard to cardiopulmonary bypass (CPB), clamping, operation, and postoperative ventilation times (Table 3).

**Table 1:** Comparison of Distribution in Demographic Variables in Three Groups

Variables	Groups				P value
		Midazolam ( N=20 )	Propofol ( N=20 )	Isoflurane ( N=20 )	
**Gender**	Male	11 ( 55% )	14 ( 70% )	16 ( 80% )	0.231
	Female	9 ( 45% )	6 ( 30% )	4 ( 20% )	
**Age groups ( year )**	< 50	1 ( 5% )	5 ( 25% )	3 ( 15% )	0.187
	51-60	12 ( 60% )	5 ( 25% )	8 ( 40% )	
	>=61	7 ( 35% )	10 ( 50% )	9 ( 45% )	
BMI*	19-25	3 ( 15% )	5 ( 25% )	4 ( 20% )	0.925
	25-30	11 ( 55% )	11 ( 55% )	11 ( 55% )	
	>30	6 ( 30% )	4 ( 20% )	5 ( 25% )	

^*^BMI: Body mass index

**Table 2:** Comparison of Clinical Data Distribution in Relation to Risk Factors in Three Groups

Variables		Groups			P value
		Midazolam ( N=20 )	Propofol ( N=20 )	Isofluran ( N=20 )	
**Hyperlipidemia**	Yes	12 ( 60% )	14 ( 70% )	9 ( 45% )	0.272
	No	8 ( 40% )	6 ( 30% )	11 ( 55% )	
**COPD** [Table-fn fn5149]	Yes	1 ( 5% )	7 ( 35% )	3 ( 15% )	0.044
	No	19 ( 95% )	13 ( 65% )	9 ( 85% )	
**High Blood Pressure**	Yes	14 ( 70% )	11 ( 55% )	8 ( 44% )	0.162
	No	6 ( 30% )	9 ( 45% )	12 ( 60% )	
**DM** [Table-fn fn5149]	Yes	9 ( 45% )	12 ( 60% )	3 ( 15% )	0.013
	No	11 ( 55% )	8 ( 40% )	17 ( 85% )	
**MI** [Table-fn fn5149]	Yes	2 ( 10% )	4 ( 20% )	4 ( 20% )	0.619
	No	18 ( 90% )	16 ( 80% )	16 ( 80% )	
**Smoking**	Yes	1 ( 5% )	6 ( 30% )	3 ( 15% )	0.102
	No	19 ( 95% )	14 ( 70% )	17 ( 85% )	

COPD= Chronic Obstructive pulmonary disease; DM= Diabetic mellitus; MI= Myocardial Infarction

**Table 3:** Mean ± SD of Measured Parameters among Three Groups

Variables	Groups			P value
	Midazolam ( N=20 )	Propofol ( N=20 )	Isofluran ( N=20 )	
**EF ( % )** [Table-fn fn5155]	50.75 ± 4.06	51.25 ± 6.46	50.75 ± 4.06	0.554
**Clamping Time (min)**	40.25 ± 8.81	42.20± 9.97	38.90 ± 6.86	0.438
**CPB Time (min)** [Table-fn fn5155]	63.45 ± 10.91	64.60 ± 16.38	60.25 ± 10.40	0.544
**Ventilation Time (min)**	506.25 ± 32.30	185.25 ± 28.80	241.25 ± 3.93	0.321

EF: Ejection fraction; CPB: Cardiopulminary by pass

There were no statistically significant differences in mean cTnT levels between three groups in the preoperative period and 12-24 hours after arrival in ICU (Table 4). However, mean cTnT in 3 groups at 36 hours after arrival in ICU were different (*P*< 0.013) and cTnT level was significantly higher in midazolam than propofol and Isoflurane groups (*P*<0.001) and lowest in patients receiving isoflurane (*P*=0.002) (Table 5).

**Table 4:** Mean ± SD of Measured cTnT Levels in Every Different Intervals Time by Three Groups of Patients

Time intervals	Groups			P value
	Midazolam ( N=20 )	Propofol ( N=20 )	Isofluran ( N=20 )	
**Pre operation**	0.02 ± 0.022	0.013 ± 0.011	0.026 ± 0.022	0.014
**12 hour** **	0.26 ± 0.17	0.20 ± 0.11	0. 14 ± 0. 15	0.072
**24 hour**	0.22 ± 0.19	0.19 ± 0.12	0.13 ± 0.17	0.277
**36 hour**	0.24 ± 0.21	0.15 ± 0. 11	0.10 ± 0.09	0.013

^**^12, 24 and 36 hour after surgery

**Table 5:** Mean ± SD of cTnT Levels at Different Time Intervals in Separate Groups

Groups	Time intervals				P value	P value
	Pre-operation	12 hour	24 hour	36 hour	Within groups*	Between groups**
**Midazolam ( N=20 )**	0.02 ± 0.022	0.26 ± 0.17	0.22 ± 0.19	0.24 ± 0.21	P<0.001*	P=0.033 **
**Propofol ( N=20 )**	0.013 ± 0.011	0.20 ± 0.11	0.19 ± 0.12	0.15 ± 0.11	P<0.001*	
**Isoflurane ( N=20 )**	0.026 ± 0.022	0.14 ± 0.15	0.13 ± 0.17	0.10 ± 0.09	P=0.002 *	

^*^ Statistically Significant (P < 0.05) for effect on time in every groups by Repeated Measurement;

^**^Statistically significant (P < 0.05) for trend analysis by Repeat Measurement;

**Figure 1:**
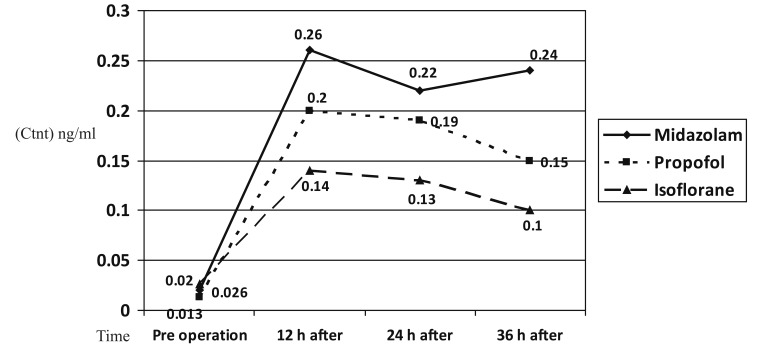
Comparison of Mean of Measured cTnT Levels at Different Time Intervals in Separate Groups

The peak level of cTnT in all three groups was detected 12 hours after arrival in ICU (Figure. 1).

## Discussion

CABG imposes some degrees of cardiac damages due to the intermittent periods of ischemia which become obvious after the surgery (2,3,7,11,17). Cardiopulmonary bypass has been reported to be associated with higher levels of myocardial necrosis compared with off- pump cardiac procedures (18,19). This has been explained to occur as a result of a prolonged disseminated ischemia as the patient goes on pump, a condition which ultimately lead to unfavorable patient’s prognosis (18,19). However, advances in surgical and anesthetic techniques are promising in avoiding undesirable outcomes (9,10,14-16, 19). In our study, all the patients were similar in terms of primary and clinical characteristics while the same surgical and anesthetic approaches were performed in all groups which only differed in regard to the agent employed for maintenance of anesthesia.

Following myocardial injury, there is a biphasic pattern of cTnT release including early phase at 3-5 h and late stage on the fifth day after cell death (4-6, 20). As this marker is not released by skeletal muscle and is secreted only in minimal concentrations at baseline, it is considered as an ideal marker to represent cardiac cell damage. Previous studies have extensively pronounced the direct relationship between cTnT and cell necrosis, apoptosis in ischemic- reperfusion models (4-6,14,16,18,19).

Previous experiments both on animal models (21-22) and among human subjects (8, 10, 15-16) are in vast majority of cases in favor of protective effects of inhaled isoflurane or intravenous administration of propofol. Propofol has been proposed to play as a scavenger in oxidative stress evoked by ischemic- reperfusion injuries during and after cardiac surgeries (21,23). However, isoflurane was shown to act via several mechanisms namely potassium-adenosine triphosphate channel opening. Subsequent cardioprotection against myocardial stunning is highly compelling (24,25). Although discrete mechanisms had been explained for either of these agents, their combination has not revealed additive results (16). However, either of these agents mimics the preconditioning effects by providing a defensive barrier for myocardium.

TAO et al. have compared the role of anesthetic preconditioning by propofol and/or isoflurane in rats under ischemia-reperfusion injury and demonstrated that these agents similarly share protection potential against myocardial ischemic injuries (26). However, combination of these two did not add to the preconditioning effects. Furthermore, studies on human subjects have not reported cardioprotective effects for propofol (16). In spite of statistical differences in distribution of D.M and COPD in three groups (Table 2) we could not find any relationship between Diabetic mellitus (DM) and chorinc obstructive pulmonary disease and variation of cTnT. Limited human studies have reported relationships between anesthetic techniques and serum levels of cTnT (8,9,14,16,21). In a pilot study, Keandall et al. showed that levels of cTnT were lowest in the propofol group followed by isoflurane and epidural anesthetic groups (16). Although not proved to be significant, their results evoked the merit of further investigations. Finally, our results showed that there was a significant difference between anesthetic groups in terms of cTnT levels only 36 h after the surgery when the amount of cTnT was lowest for isoflurane followed by propofol and midazolam groups. Furthermore, in early stages, patients of midazolam group had the highest level of cTnT while the lowest values varied between propofol and isoflurane groups. Besides, the difference between the slopes of cTnT changes ,although more vertical in midazolam compared to propofol and isoflurane groups, was not statistically significant.

Our results revealed a significant difference in cTnT levels among anesthetic groups of isoflurane, midazolame, and propofol at 36th hours after surgery. However, the differing values were not significant at other time intervals.

Finally one of the limitations of our study was the differences in incidence of diabetes mellitus and COPD in 3 groups, and further studies are recommended to determine the effects of these diseases on cTnT levels in CABG patients.
